# Posterior Ankle Arthroscopy for Osteochondromatosis of the Posterior Ankle Extra-Articular Space with a Longitudinal Tear of Flexor Hallucis Longus

**DOI:** 10.1155/2020/6580472

**Published:** 2020-07-06

**Authors:** Ichiro Tonogai, Koichi Sairyo

**Affiliations:** Department of Orthopedics, Institute of Biomedical Science, Tokushima University Graduate School, 3-18-15 Kuramoto, Tokushima City, Tokushima 770-8503, Japan

## Abstract

We report a rare case of osteochondromatosis of the posterior ankle extra-articular space with a longitudinal tear of flexor hallucis longus (FHL). A 77-year-old woman was referred to our hospital with an approximately 4-year history of pain and swelling in the right posterior ankle joint without obvious trauma. The pain had worsened in the previous 2 years. On presentation, she had tenderness at the posteromedial and posterolateral ankle. Imaging revealed several ossified loose bodies in the posterior ankle extra-articular space. We removed the loose bodies, performed tenosynovectomy around the FHL, and released the FHL tendon using a posterior arthroscopic technique via standard posterolateral and posteromedial portals. A longitudinal tear and fibrillation were detected in the FHL. The patient was able to return to her daily activities approximately 3 weeks after surgery. At the 1-year follow-up visit, she continued to have minor discomfort and slight swelling on the posteromedial aspect of the right ankle but had no recurrence of the ossified loose bodies. To our knowledge, this is the first report of osteochondromatosis of the posterior ankle extra-articular space with a longitudinal tear of the FHL that was treated by removal of loose bodies, tenosynovectomy around the FHL, and release of the FHL tendon via posterior ankle arthroscopy.

## 1. Introduction

Osteochondroma is the most common bone tumor and is the developmental lesion rather than the true neoplasm [1]. It is 20–50% of all benign bone tumors and 10–15% of all bone tumors [1]. Pathogenesis of osteochondroma is still controversial, but some osteochondromas can be replaced by osseous tissue through the process of enchondral ossification, and these chondromas are ossified over a period of time finally [2]. Osteochondroma is the lesion that is composed of cortical and medullary bone with an overlying hyaline cartilage cap. Among osteochondromas, osteochondroma of the talus was first reported in 1984 by Fuselier et al. [3], but posterior talar osteochondroma is rare [2, 4–8].

Longitudinal flexor hallucis longus (FHL) tendon tears are sometimes complicated by posterior ankle impingement syndrome [9]. The presence of an os trigonum has an important role in FHL pathologies, such as tenosynovitis with degenerative changes and partial tendon tear [10]. However, to date, there have been no reports on a longitudinal tear of the FHL tendon that might be caused by friction between posterior ankle osteochondroma and FHL or on endoscopic removal of loose bodies in osteochondromatosis.

Here, we report a case of osteochondromatosis of the posterior ankle extra-articular space with a longitudinal tear of the FHL that was treated by resection of loose bodies that might be caused by osteochondromatosis, tenosynovectomy around the FHL, and release of the FHL tendon via posterior ankle arthroscopy.

## 2. Case Presentation

**Figure 1:**
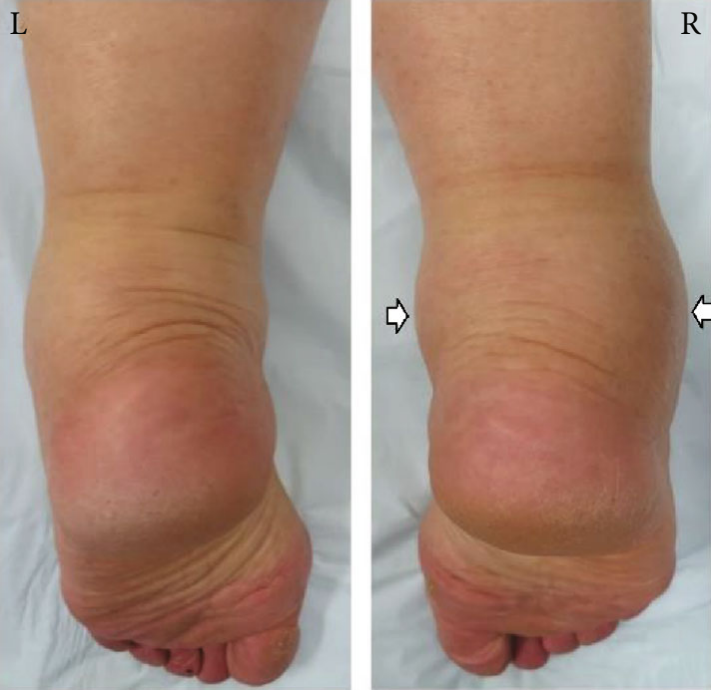
Preoperative photograph showing swelling of the posteromedial and posterolateral aspects (white arrows) of the right ankle.

**Figure 2:**
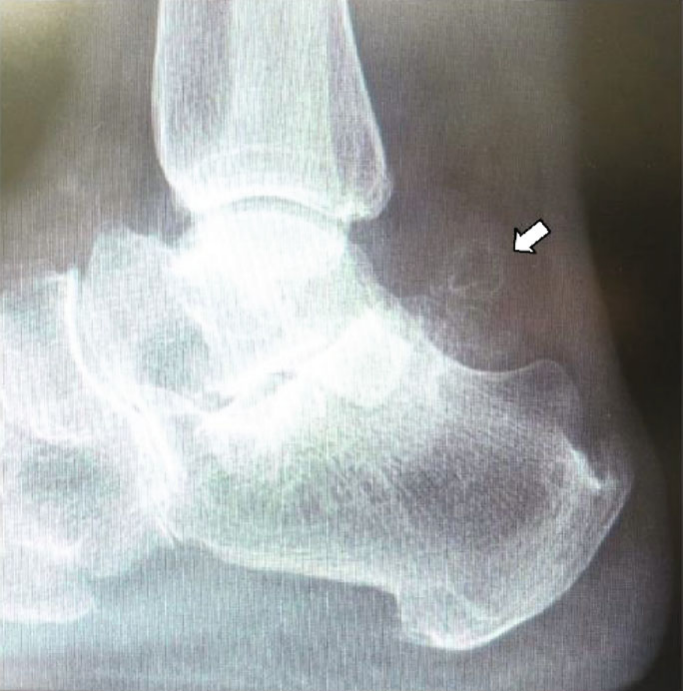
Lateral plain radiographic view of the right ankle obtained preoperatively showing several ossified masses in the posterior ankle area (arrow).

**Figure 3:**
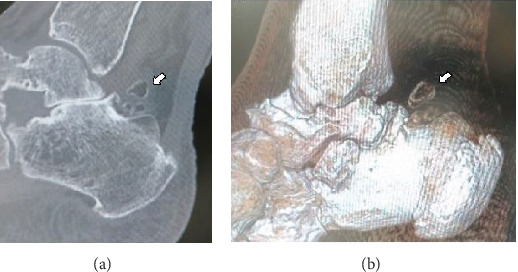
Computed tomography scans showing homogeneous calcifications with typical ring-and-arc chondroid mineralization in the posterior ankle (a) on a plain sagittal view (arrow) and (b) on a three-dimensional image view (arrow).

**Figure 4:**
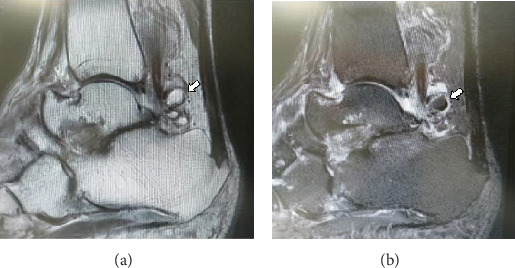
Magnetic resonance imaging shows several small fusiform masses (arrow) surrounded by a fluid signal on T2-weighted (a) and short T1 inversion recovery (b) scans in the sagittal plane.

Informed consent was obtained from the patient for this report to be published. The patient was a 77-year-old woman who was referred to our department with an approximately 4-year history of pain and swelling of the right ankle without obvious trauma. Her pain had worsened about 2 years earlier, at which time she was treated conservatively by a local doctor but her symptoms persisted. She had no past medical history. At the first visit to our department, physical examination revealed swelling and tenderness of the medial and lateral aspects of the ankle (Figure 1). There was slight limitation of range of motion of the right ankle. No neurovascular deficit was noted. Her JSSF (Japanese Society for Surgery of the Foot) scale score was 42/100 (pain 0/40, function 32/50, and alignment 10/10). The patient rated her pain as 7/10 on a numerical rating scale (NRS). A weight-bearing lateral plain radiographic view of the loose bodies in the posterior ankle extra-articular space is shown in Figure 2. Several loose bodies were seen on the ossified lesion in osteochondromatosis on computed tomography scans (Figures 3(a) and 3(b)). T2-weighted magnetic resonance images showed hypointense areas typical of hyaline cartilage peripherally in the posterior ankle space, and short T1 inversion recovery images showed hypointense areas indicating loose bodies surrounded by effusion (Figures 4(a) and 4(b)). The preoperative diagnosis was osteochondromatosis of the posterior ankle extra-articular space, and a plan was made to reduce the patient's pain surgically using a minimally invasive arthroscopic approach.

**Figure 5:**
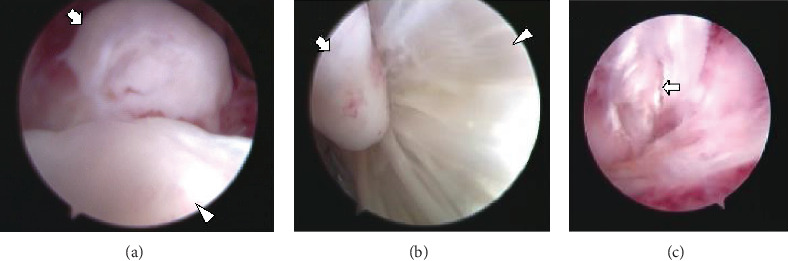
Arthroscopic views of the posterior aspect of the right ankle. (a) A large loose body from the right posterior ankle extra-articular space (arrow) can be visualized. The arrowhead follows the superior-posterior aspect of the calcaneus. (b) Friction between a loose body (arrow) and the FHL tendon (arrowhead) can be seen. (c) A longitudinal tear and fibrillation were detected in the FHL tendon (arrow). FHL: flexor hallucis longus.

**Figure 6:**
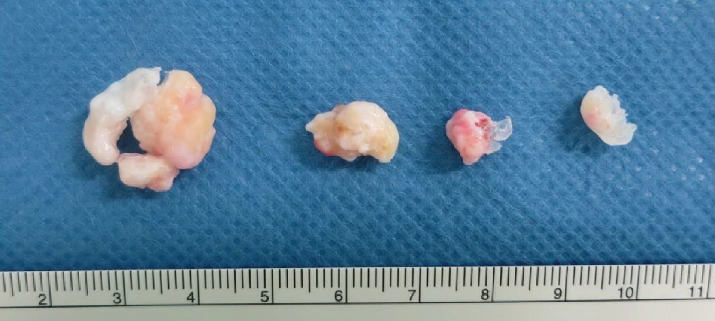
Photograph showing the loose bodies that were endoscopically removed from the right posterior ankle extra-articular space.

**Figure 7:**
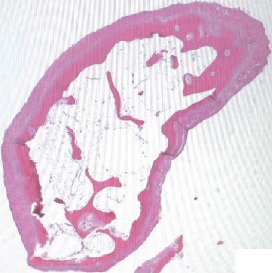
Histology slide showing peripheral chondrocytes.

The patient was positioned prone, and a thigh tourniquet was placed. Two portals were created 1 cm above the insertion of the Achilles tendon, one just medial and one just lateral to the tendon, in line with the tip of the lateral malleolus using the standard 2-portal technique described by van Dijk et al. [11]. The lateral portal was used for visualization, and the medial one was used as the working portal. A 4 mm, 30-degree arthroscope was introduced through the portals and directed towards the second toe. Several ossified loose bodies were then visualized at the posterior aspect of the talus in the posterior ankle space (Figure 5(a)). Loose bodies were impinging between the posterior aspect of the talus and calcaneus with friction between the loose bodies and the FHL tendon (Figure 5(b)). After removal of the loose bodies in the posterior ankle space, the entire FHL tendon sheath could be visualized and the tendon was released with a shaver. Fibrillation and a friction-related longitudinal tear of the FHL were seen (Figure 5(c)) but could not be treated during posterior ankle arthroscopy. We elected not to convert to an open repair. There were no intraoperative complications. In total, four loose bodies were removed endoscopically from the right posterior ankle extra-articular space (Figure 6). The histology report confirmed hypercellularity in the hyaline cartilage peripherally with no evidence of nuclear pleomorphism associated with binucleated or multinucleated lacunae, necrosis, or mitotic activity in the ossified lesion (Figure 7). Histological findings were consistent with osteochondroma, although calcifying aponeurotic fibroma, tumoral calcinosis, the focal form of pigmented villonodular synovitis, synovial sarcoma, and extraskeletal chondrosarcoma should be considered the differential diagnosis for osteochondroma. Therefore, we made a final diagnosis of osteochondroma of the posterior ankle extra-articular space.

**Figure 8:**
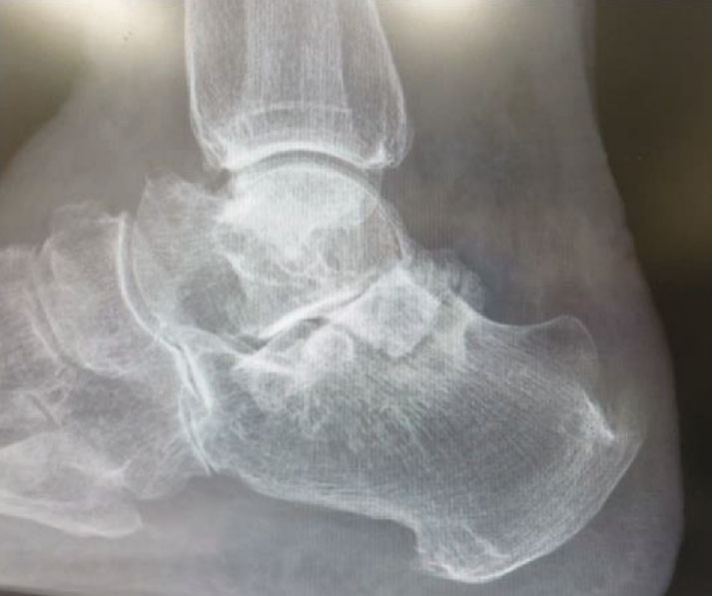
Lateral plain radiographic view of the right ankle showing no ossified loose bodies in the right posterior ankle extra-articular space.

**Figure 9:**
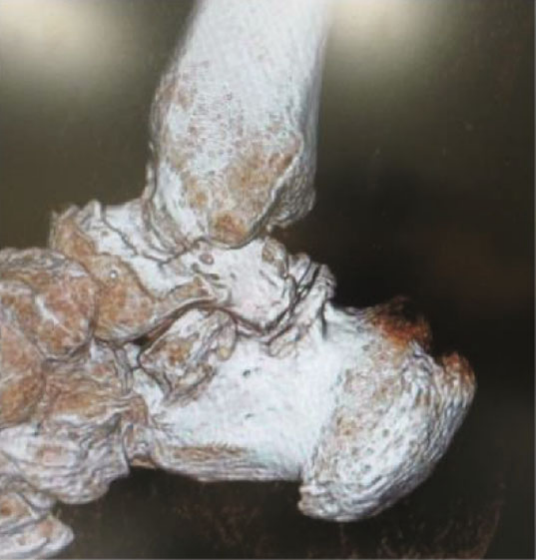
Three-dimensional computed tomography scan obtained postoperatively shows no ossified lesions in the posterior ankle.

A bulky dressing was placed postoperatively without any immobilization. The patient was encouraged to actively move her ankle and toes. Weight bearing was allowed after surgery as long as tolerated. Return to daily activities was allowed after 3 weeks. The postoperative course was unremarkable. At the 1-year follow-up visit, the patient was very satisfied with the surgery, although there was some discomfort and slight swelling on the right posteromedial aspect of the ankle. Plain radiographic and three-dimensional computed tomographic images showed no recurrence of the ossified masses (Figures 8 and 9). Her JSSF scale score improved from 42/100 to 90/100 (pain 30/40, function 50/50, and alignment 10/10), and her NRS improved to 1/10.

## 3. Discussion

We treated a 77-year-old woman who presented with osteochondromatosis of the posterior ankle extra-articular space accompanied by a longitudinal tear of the FHL by resection of the loose bodies that might be caused by osteochondromatosis, tenosynovectomy around the FHL, and release of the FHL tendon via posterior ankle arthroscopy. There are a few reports on osteochondromatosis in the posterior extra-articular ankle space treated via a posterior ankle arthroscopic approach [2, 6, 7] but none that include a longitudinal tear of the FHL that might be caused by friction between the loose bodies and the FHL, as this case.

The FHL tendon passes through a tendon sheath arising from the flexor retinaculum at the posterior talus into a fibro-osseous tunnel along the medial calcaneus and inferior aspect of the sustentaculum tali. There has been a report suggesting that the passage of loose bodies along the tendon sheath can be facilitated by the gliding motion of the FHL tendon during walking and that FHL tenosynovitis can be secondary to irritation by the loose bodies [7]. Consistent with that report, the longitudinal tear and fibrillation of the FHL in our patient seemed to be caused by irritation from the loose bodies of osteochondromatosis.

Hamilton et al. reported 5 cases of longitudinal tears in the FHL among dancers with pain in the posterior aspect of the ankle who underwent open surgery, including one patient in whom a diagnosis of probable partial intratendinous rupture was made on the basis of a widened external appearance [12]. However, their series did not include any cases of osteochondromatosis in the posterior ankle, as in our patient. Hamilton et al. also reported finding only one longitudinal tear of the FHL in 28 unselected cadaveric ankles from elderly patients [12]. Given that our patient was also elderly, the possibility that the longitudinal tear and fibrillation of the FHL predated the friction between the loose bodies and the FHL cannot be excluded. Moreover, the postulation about tear at FHL was solely due to friction between it and the loose bodies would be challenged.

Open surgery for osteochondromatosis in the posterior ankle space has been reported [8, 13]. However, there are few reports on posterior arthroscopic surgery for osteochondromatosis in the posterior ankle space [2, 6, 7]. Arthroscopic approaches have gained popularity in the past decade because they cause less scarring and postoperative pain, are associated with minimal overall morbidity, and allow earlier return to normal activity. Improved visualization of pathology, less postoperative pain, a decreased risk of complications, and rapid return to physical activity have been specifically mentioned as advantages of the endoscopic approach to the posterior hindfoot and ankle [14]. Therefore, we selected an ankle arthroscopic procedure to avoid the need for extensive soft tissue dissection required with open surgery. The clinical results in our case confirm the effectiveness of a posterior ankle arthroscopy approach for removal of loose bodies that might be caused by osteochondromatosis in the posterior ankle space.

One limitation of this report is the short follow-up duration. Although there was no recurrence of ossified masses at the most recent follow-up visit 1 year after surgery, further follow-up is necessary. Another limitation is that we did not treat the longitudinal tear and fibrillation of the FHL by posterior ankle arthroscopy because we considered that it would be too difficult to suture an FHL tendon tear endoscopically, despite a previous report to the contrary by Nishimura et al. [9]. However, our patient's complaints of minor discomfort and slight painless swelling in the right posteromedial ankle at the 1-year follow-up could have been caused by the longitudinal tear and fibrillation in the FHL. Therefore, follow-up is ongoing to determine if further surgery for the FHL is necessary. Another limitation is that we have no histology of the synovium. We should have obtained a sample of the synovium for histological examination. Another limitation is this patient seems to have talocalcaneal osteoarthritis from image findings. Some authors reported that osteoarthritis can coexist in some cases [15–17]. Therefore, there might be a possibility that free bodies of this patient originated from osteoarthritis.

In conclusion, we encountered a rare case of osteochondromatosis of the posterior ankle extra-articular space accompanied by a longitudinal tear in the FHL that might be caused by friction between the loose bodies and the FHL. The patient was treated by resection of the loose bodies that might be caused by osteochondromatosis, tenosynovectomy around the FHL, and release of the FHL tendon via a posterior ankle arthroscopy.
